# Medical Association of Georgia

**Published:** 1867-05

**Authors:** 


### MEDICAL ASSOCIATION OF GEORGIA.

We had the pleasure of attending the meeting of this body, in Griffin, on the 10th inst.

The number in attendance, even in the embarrassed condition of the country, was larger than usual before the war.

The meeting was harmonious, and members exhibited a disposition to make the Association more useful to the country and the profession, than heretofore. Instead of passing through the session, with no other business than the discussion of questions connected with the government of the body, &c., there seemed to be a decided disposition in several of the members, (to their praise be it said) to present discoveries and improvements in practical medicine. These certainly should be the cheif objects of the organization. The subjects presented, and the discussions upon them at the recent meeting, were quite interesting. We hope to hear a still larger number of reports at the next anual session, particularly on the recently discovered medicinal properties of indigenous plants. We give below the proceedings of the Association:

